# RNA-Seq Based Transcriptional Map of Bovine Respiratory Disease Pathogen “*Histophilus somni* 2336”

**DOI:** 10.1371/journal.pone.0029435

**Published:** 2012-01-20

**Authors:** Ranjit Kumar, Mark L. Lawrence, James Watt, Amanda M. Cooksey, Shane C. Burgess, Bindu Nanduri

**Affiliations:** 1 College of Veterinary Medicine, Mississippi State University, Mississippi State, Mississippi, United States of America; 2 Institute for Genomics, Biocomputing and Biotechnology, Mississippi State University, Mississippi State, Mississippi, United States of America; 3 Center for Clinical and Translational Science, University of Alabama at Birmingham, Birmingham, Alabama, United States of America; 4 Eagle Applied Sciences LLC, San Antonio, Texas, United States of America; 5 College of Agriculture and Life Sciences, The University of Arizona, Tucson, Arizona, United States of America; Kyushu Institute of Technology, Japan

## Abstract

Genome structural annotation, i.e., identification and demarcation of the boundaries for all the functional elements in a genome (e.g., genes, non-coding RNAs, proteins and regulatory elements), is a prerequisite for systems level analysis. Current genome annotation programs do not identify all of the functional elements of the genome, especially small non-coding RNAs (sRNAs). Whole genome transcriptome analysis is a complementary method to identify “novel” genes, small RNAs, regulatory regions, and operon structures, thus improving the structural annotation in bacteria. In particular, the identification of non-coding RNAs has revealed their widespread occurrence and functional importance in gene regulation, stress and virulence. However, very little is known about non-coding transcripts in *Histophilus somni*, one of the causative agents of Bovine Respiratory Disease (BRD) as well as bovine infertility, abortion, septicemia, arthritis, myocarditis, and thrombotic meningoencephalitis. In this study, we report a single nucleotide resolution transcriptome map of *H. somni* strain 2336 using RNA-Seq method.

The RNA-Seq based transcriptome map identified 94 sRNAs in the *H. somni* genome of which 82 sRNAs were never predicted or reported in earlier studies. We also identified 38 novel potential protein coding open reading frames that were absent in the current genome annotation. The transcriptome map allowed the identification of 278 operon (total 730 genes) structures in the genome. When compared with the genome sequence of a non-virulent strain 129Pt, a disproportionate number of sRNAs (∼30%) were located in genomic region unique to strain 2336 (∼18% of the total genome). This observation suggests that a number of the newly identified sRNAs in strain 2336 may be involved in strain-specific adaptations.

## Introduction

Systems biology approaches are designed to facilitate the study of complex interactions among genes, proteins, and other genomic elements [1], [2], [3]. In the context of infectious disease, systems biology has the potential to complement reductionist approaches to resolve the complex interactions between host and pathogen that determine disease outcome. However, a prerequisite for systems biology is the description of the system's components. Therefore, genome structural annotation or the identification and demarcation of boundaries of functional elements in a genome (e.g., genes, non-coding RNAs, proteins, and regulatory elements) are critical elements in infectious disease systems biology.

Bovine Respiratory Disease (BRD) costs the cattle industry in the United States as much as $3 billion annually [4], [5]. BRD is the outcome of complex interactions among host, environment, bacterial, and viral pathogens [6]. *Histophilus somni*, a gram-negative, pleomorphic species, is one of the important causative agents of BRD [6]. *H. somni* causes bovine infertility, abortion, septicemia, arthritis, myocarditis, and thrombotic meningoencephalitis [7]. *H. somni* strain 2336, the serotype used in this study and isolated from pneumonic calf lung, has a 2.2 Mbp genome and 2044 predicted open reading frames (ORFs), of which 1569 (76%) have an assigned biological function.

Genome structural annotation is a multi-level process that includes prediction of coding genes, pseudogenes, promoter regions, repeat elements, regulatory elements in intergenic regions such as small non-coding RNAs (sRNA), and other genomic features of biological significance. Computational gene prediction methods such as Glimmer [8] or GenMark [9] use Hidden Markov models which are based on a training set of well annotated genes. Although these methods are quite efficient, they often miss genes with anomalous nucleotide composition and have several well-described shortcomings: because bacterial genomes do not have introns, detecting gene boundaries is comparatively difficult; due to the usage of more than one start codon, computational genome annotation methods may predict overlapping ORFs [10]; prediction programs use arbitrary minimum cutoff lengths to filter short ORFs, which may lead to under-representation of small genes. In case of sRNA (small non-coding RNA) prediction, the lack of DNA sequence conservation, lack of a protein coding frame, and the limited accuracy of transcriptional signal prediction programs (promoter/Rho terminator prediction) confound computational prediction [11], [12].

Computational prediction methods are a “first pass” genome structural annotation. Whole genome transcriptome studies (such as whole genome tiling arrays [13], [14], [15] and high throughput sequencing [16], [17]) are complementary experimental approaches for bacterial genome annotation and can identify “novel” genes, gene boundaries, regulatory regions, intergenic regions, and operon structures. For example, a transcriptomic analysis of *Mycoplasma pneumoniae* identified 117 previously unknown transcripts, many of which were non-coding RNAs, and two novel genes [18]. Transcriptome analyses identified novel, non-coding regions in other species, including 27 sRNAs in *Caulobacter crescentus* [15], 64 sRNAs in *Salmonella Typhimurium* [17], and a large number of putative sRNAs in *Vibrio cholerae* [16]. sRNAs found in pathogen genomes are known to be involved in various housekeeping activities and virulence [19].

In this study we used RNA-Seq for the experimental annotation of the *H. somni* strain 2336 genome and to construct a single nucleotide resolution transcriptome map. Novel expressed elements were identified, and where appropriate, computational predictions of previously described gene boundaries were corrected.

## Results

### Mapping of reads onto the *H. somni* genome

In 2008 the complete genome sequence of the *H. somni* strain 2336 became available (GenBank CP000947). The 2,263,857 bp circular genome has a GC content of 37.4%, and 87% of the sequence is annotated to coding regions. The genome has 2065 computationally predicted genes, of which 1980 are protein coding. We sequenced the transcriptome of *H. somni* using Illumina RNA-Seq methodology, and obtained 9,015,318 reads, with an average read length of approximately 76 bp. We mapped approximately 9.4% reads onto the reference DNA sequence of *H. somni* strain 2336 using the alignment program Bowtie [20]. To determine expressed regions in the genome, we estimated the average coverage depth of reads mapped per nucleotide/base. We used pileup format, which represents the signal map file for the whole genome in which alignment results (coverage depth) are represented in per-base format. Regions where coverage depth was greater than the lower tenth percentile of expressed genes were considered significantly expressed [21]; in the current study, this corresponded to a coverage depth of 7 reads/bp in pileup format.

**Figure 1:**
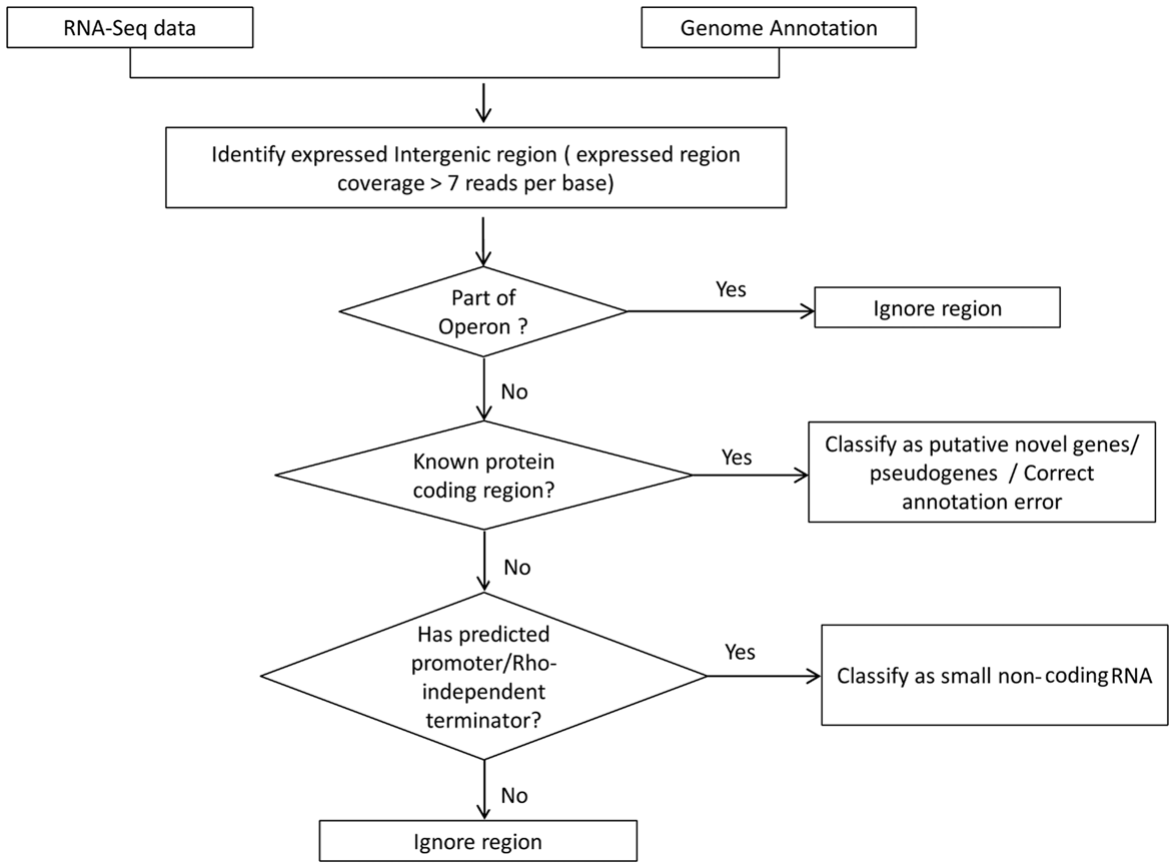
RNA-Seq data analysis workflow for intergenic expression analysis. Analysis workflow includes identification of novel protein coding genes and sRNAs in the intergenic region of *H. somni* 2336 genome.

As another measure for estimating background expression level, we analyzed the coverage in the intergenic regions of the genome. We assumed that at least half of the intergenic region is not expressed (considering the presence of known expressed regions, such as 3′ and 5′ UTR of genes, intergenic region of the operons, and sRNAs) and calculated the coverage, which corresponded to ≤6 reads per base, lower than our first cutoff estimate. We retained the most conservative cutoff for expression, i.e., 7 reads per base for describing the expression map of *H. somni*. Nucleotides in the genome sequence with coverage depth above our threshold value were considered to be expressed. This resulted in the generation of a whole genome transcriptome profile of *H. somni 2336* at a single nucleotide resolution. Figure 1 show the steps involved in the analysis of expressed intergenic regions.

### Expression in the intergenic region of the genome

**Figure 2:**
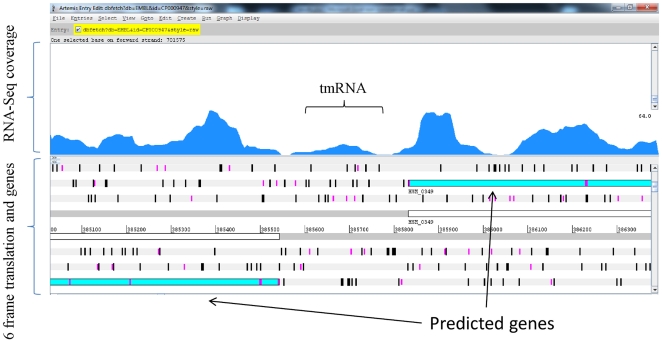
Identification of sRNA annotated to Rfam. The figure shows identification of well conserved sRNA “tmRNA” using RNA-Seq based method. “tmRNA” was computationally predicted as a sRNA by Rfam using sequence similarity across other bacterial families.

**Figure 3:**
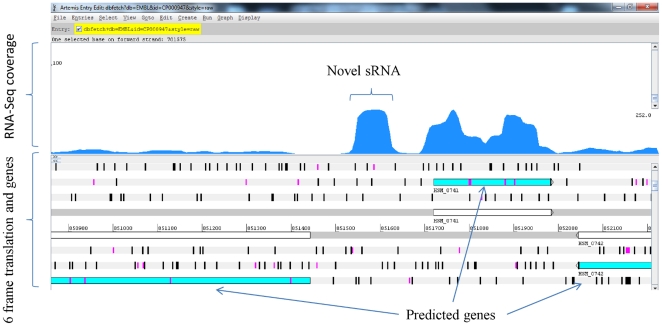
Identification of a novel sRNA. A highly expressed sRNA “HS46” found in the intergenic region of *H. somni* 2336 genome.

We compared the RNA-Seq based transcriptome map with the available genome annotation to identify expressed, novel, and intergenic regions in the genome. Promoters and terminators were predicted across the genome to add confidence to the identified novel elements. For the first time, we report the identification of 94 sRNAs (Table 1) in the *H. somni* genome. The start and end for sRNA in Table 1 refer to the boundaries of transcriptionally active regions (TAR, putative sRNAs). Of these, twelve were similar to well-characterized sRNA families that are described in many bacterial species, such as tmRNA, 6S, and FMN (Figure 2). The total of 82 novel sRNAs reported in this study has not been reported earlier. The majority of the identified sRNAs (>75%) were shorter than 200 nucleotides (length range 70–695 nucleotides). The average GC content of sRNA at 39.3% was slightly higher compared with the 37.4% GC content of the genome. Promoters within 50 nt upstream/downstream of the TAR boundaries were predicted for 68 sRNA. Similarly, Rho-independent transcription terminators were predicted within 50 bp upstream/downstream of 40 sRNA. Figure 3 shows the depth of coverage for one of the identified novel sRNA “HS46” viewed in the Artemis genome browser [22].

**Table 1:** *H. Somni* 2336 sRNAs, their genome location, additional features and comparative genomics.

ID	Start#	End#	Length (nt)	Promoter	Rho independent terminator	Flanking gene (left)	Flanking gene(right)	Rfam annotation	Conservation across other genome*
HS1	8109	8210	101	-	Y	HSM0009 (+)	HSM0010 (+)	-	C
HS2	16119	16190	71	Y	Y	HSM0018 (+)	HSM0019 (−)	-	B
HS3	27693	27843	150	Y	-	HSM0034 (+)	HSM0035 (+)	-	B
HS4	28211	28327	116	Y	-	HSM0035 (+)	HSM0036 (+)	-	B
HS5	29449	29733	284	Y	-	HSM0037 (+)	HSM0038 (+)	-	C
HS6	30644	30884	240	Y	Y	HSM0039 (+)	HSM0040 (−)	-	B
HS7	91913	92041	128	-	Y	HSM0081 (−)	HSM0082 (+)	-	C
HS8	113922	114088	166	Y	Y	HSM0102 (+)	HSM0103 (−)	-	C
HS9	161819	161939	120	Y	-	HSM0149 (+)	HSM0150 (+)	-	B
HS10	183097	183180	83	Y	-	HSM0171 (−)	HSM0172 (+)	-	B
HS11	197465	197654	189	-	-	HSM0185 (−)	HSM0186 (−)	-	B
HS12	229676	229780	104	Y	-	HSM0213 (+)	HSM0214 (−)	-	C
HS13	243592	243683	91	-	Y	HSM0224 (+)	HSM0225 (−)	-	A
HS14	258140	258443	303	Y	Y	HSM0242 (−)	HSM0243 (−)	-	A
HS15	258962	259092	130	Y	Y	HSM0244 (−)	HSM0245 (+)	-	A
HS16	260385	260577	192	Y	Y	HSM0245 (+)	HSM0246 (−)	-	A
HS17	261314	261511	197	-	Y	HSM0246 (−)	HSM0247 (−)	-	A
HS18	261829	262158	329	Y	Y	HSM0246 (−)	HSM0247 (−)	-	A
HS19	264263	264362	99	Y	-	HSM0250 (+)	HSM0251 (+)	-	A
HS20	279541	279733	192	-	-	HSM0266 (−)	HSM0267 (+)	-	B
HS21	306503	306957	454	Y	-	HSM0284 (+)	HSM0285 (−)	tmrna	D
HS22	318188	318443	255	-	-	HSM0292 (+)	HSM0293 (−)	-	D
HS23	319335	319635	300	-	-	HSM0295 (+)	HSM0296 (+)	-	B
HS24	341911	342015	104	-	-	HSM0316 (−)	HSM0317 (−)	-	B
HS25	343637	343745	108	-	-	HSM0318 (−)	HSM0319 (+)	-	B
HS26	377953	378037	84	Y	-	HSM0345 (+)	HSM0346 (−)	-	B
HS27	383294	383421	127	Y	-	HSM0346 (−)	HSM0347 (+)	-	A
HS28	385627	385733	106	Y	Y	HSM0348 (−)	HSM0349 (+)	-	B
HS29	391757	392003	246	-	-	HSM0353 (−)	HSM0354 (−)	-	C
HS30	412425	412522	97	-	-	HSM0368 (−)	HSM0369 (−)	-	B
HS31	472256	472331	75	Y	Y	HSM0407 (−)	HSM0408 (−)	-	B
HS32	524433	524805	372	Y	-	HSM0448 (+)	HSM0450 (−)	-	A
HS33	599224	599416	192	Y	Y	HSM0521 (−)	HSM0522 (+)	-	B
HS34	614906	615013	107	-	-	HSM0538 (+)	HSM0539 (−)	intron_gpII	A
HS35	616791	617486	695	Y	-	HSM0539 (−)	HSM0540 (−)	-	A
HS36	617726	618078	352	Y	-	HSM0539 (−)	HSM0540 (−)	-	A
HS37	618122	618228	106	Y	Y	HSM0539 (−)	HSM0540 (−)	-	A
HS38	637931	638076	145	Y	Y	HSM0552 (+)	HSM0553 (+)	-	B
HS39	638222	638366	144	-	-	HSM0552 (+)	HSM0553 (+)	-	B
HS40	653813	653962	149	Y	-	HSM0561 (+)	HSM0562 (−)	-	A
HS41	694580	694680	100	Y	-	HSM0594 (−)	HSM0595 (−)	-	A
HS42	703333	703423	90	Y	Y	HSM0607 (+)	HSM0608 (+)	-	B
HS43	710363	710450	87	Y	-	HSM0611 (+)	HSM0612 (+)	-	B
HS44	747233	747386	153	Y	-	HSM0644 (−)	HSM0645 (−)	-	A
HS45	800181	800295	114	Y	Y	HSM0704 (+)	HSM0705 (+)	-	B
HS46	851529	851662	133	Y	Y	HSM0740 (−)	HSM0741 (+)	-	B
HS47	853988	854118	130	Y	-	HSM0742 (−)	HSM0743 (+)	-	B
HS48	876355	876433	78	-	-	HSM0758 (+)	HSM0759 (+)	glycine	C
HS49	979462	979681	219	Y	-	HSM0844 (+)	HSM0845 (+)	-	B
HS50	981925	982225	300	-	-	HSM0847 (+)	HSM0848 (+)	-	C
HS51	994234	994314	80	Y	-	HSM0853 (+)	HSM0854 (+)	-	B
HS52	1007799	1008007	208	Y	Y	HSM0868 (−)	HSM0869 (−)	-	C
HS53	1008086	1008580	494	-	Y	HSM0868 (−)	HSM0869 (−)	-	A
HS54	1012617	1012823	206	-	Y	HSM0874 (−)	HSM0875 (−)	-	B
HS55	1014425	1014768	343	Y	-	HSM0875 (−)	HSM0876 (−)	-	A
HS56	1015189	1015390	201	Y	-	HSM0877 (+)	HSM0878 (+)	-	A
HS57	1021919	1022474	555	Y	-	HSM0888 (−)	HSM0889 (−)	-	A
HS58	1031980	1032132	152	Y	Y	HSM0900 (+)	HSM0901(+)	-	C
HS59	1032206	1032458	252	Y	Y	HSM0900 (+)	HSM0901 (+)	-	D
HS60	1052587	1052754	167	Y	-	HSM0920 (+)	HSM0921 (+)	-	A
HS61	1147201	1147290	89	Y	-	HSM1005 (+)	HSM1006 (+)	-	B
HS62	1260621	1260860	239	-	-	HSM1095 (+)	HSM1096 (+)	6 s	D
HS63	1292413	1292563	150	Y	-	HSM1125 (−)	HSM1126 (+)	-	B
HS64	1307757	1307987	230	Y	-	HSM1136 (+)	HSM1137 (−)	-	A
HS65	1312693	1312855	162	Y	Y	HSM1143 (+)	HSM1144 (+)	-	A
HS66	1320228	1320349	121	Y	Y	HSM1155 (−)	HSM1156 (−)	-	A
HS67	1337412	1337590	178	Y	Y	HSM1172 (+)	HSM1173 (+)	-	B
HS68	1343583	1343659	76	Y	Y	HSM1182 (+)	HSM1183 (+)	-	D
HS69	1377309	1377411	102	Y	-	HSM1218 (+)	HSM1219 (+)	-	C
HS70	1413741	1413887	146	-	-	HSM1254 (−)	HSM1255 (+)	lysine	B
HS71	1455529	1455708	179	Y	-	HSM1275 (−)	HSM1276 (−)	MOCORNA	B
HS72	1513886	1513955	69	Y	-	HSM1330 (+)	HSM1331 (+)	-	B
HS73	1537168	1537267	99	-	-	HSM1355 (−)	HSM1356 (−)	LR-PK1	B
HS74	1591107	1591187	80	Y	Y	HSM1392 (−)	HSM1393 (−)	-	B
HS75	1593953	1594392	439	Y	-	HSM1395 (−)	HSM1396 (+)	RNaseP_bact_a	D
HS76	1596011	1596138	127	Y	Y	HSM1397 (+)	HSM1398 (+)	-	B
HS77	1748563	1748820	257	Y	Y	HSM1521 (−)	HSM1522 (+)	-	D
HS78	1752653	1752795	142	Y	-	HSM1525 (+)	HSM1526 (+)	-	A
HS79	1839524	1839616	92	Y	Y	HSM1590 (−)	HSM1591 (+)	-	B
HS80	1859168	1859317	149	Y	Y	HSM1612 (−)	HSM1613 (−)	-	B
HS81	1874398	1874609	211	Y	Y	HSM1626 (+)	HSM1627 (+)	isrK	B
HS82	1925814	1925932	118	-	-	HSM1675 (−)	HSM1676 (−)	-	B
HS83	1927797	1928029	232	Y	-	HSM1676 (−)	HSM1677 (+)	-	D
HS84	1928157	1928331	174	Y	Y	HSM1676 (−)	HSM1677 (+)	-	A
HS85	1942445	1942617	172	Y	Y	HSM1692 (+)	HSM1693 (+)	-	A
HS86	1962487	1962618	131	Y	Y	HSM1719 (−)	HSM1720 (−)	-	A
HS87	2020545	2020668	123	-	-	HSM1776 (−)	HSM1777 (+)	gcvB	D
HS88	2124794	2124884	90	Y	Y	HSM1868 (−)	HSM1869 (−)	-	A
HS89	2136245	2136324	79	Y	-	HSM1881 (−)	HSM1882 (−)	-	A
HS90	2139563	2139823	260	Y	Y	HSM1887 (+)	HSM1888 (+)	-	A
HS91	2146286	2146459	173	-	-	HSMR0065 (+)	HSM1893 (+)	-	B
HS92	2210148	2210318	170	-	-	HSM1950 (+)	HSM1951 (+)	-	B
HS93	2223802	2223946	144	-	-	HSM1974 (+)	HSM1975 (+)	alpha_RBS	D
HS94	2229269	2229450	181	-	-	HSM1982 (−)	HSM1983 (+)	FMN	D

*sRNA sequences conserved in; A - unique to *H. somni* 2336. B - *H. somni* strain 129PT only. C – phylogenetically closer bacterial genomes specially members of *Pasteurellaceae* family (*M. haemolytica, P. multocida. H. influenza* etc). D - across distant bacterial species.

# The start and end represents the boundaries of identified TAR (transcriptionally active region) which is a potential sRNA region.

Any cell with no predicted result is marked with ‘−’.

BLAST analysis of the sRNA sequences against the non-redundant, nucleotide database at NCBI revealed that 31 of the sRNA sequences were unique to the *H. somni* 2336 genome. Another 41 were highly conserved (>95% identity with >95% coverage) only in *H. somni* strain 129PT, which is a commensal, preputial isolate. A set of 11 sRNAs were conserved in the related *Pasteurellaceae* family, which includes genomes such as *P. multocida, H. influenzae, H. parainfluenzae,* and *H. ovis*. Only 11 sRNAs were conserved in distant bacterial genomes from genera *Streptococcus, Clostrodium, Actinobacillus, Vibrio,* and others. This lack of sRNA sequence conservation beyond the species could indicate that sRNA sequences are under strong selection pressure, and that they could be responsible for the adaptation of many species to different environmental niches.

We searched all *H. somni* sRNA sequences against the Rfam database [23] to determine their putative functions. We found that 12 sRNAs were homologs to well characterized sRNAs in other genomes. The identified functional categories included FMN riboswitches, gcvB, glycine, intron_gpII, lysine, alpha_RBS, LR-PK1, isrK, MOCORNA, RNaseP_bact_a, tmRNA, and 6S. sRNAs for which no Rfam function could be predicted represent a completely novel set of non-coding sRNAs. Functions of these novel sRNA need to be determined by further experiments.

### Identification and characterization of novel genes

**Figure 4:**
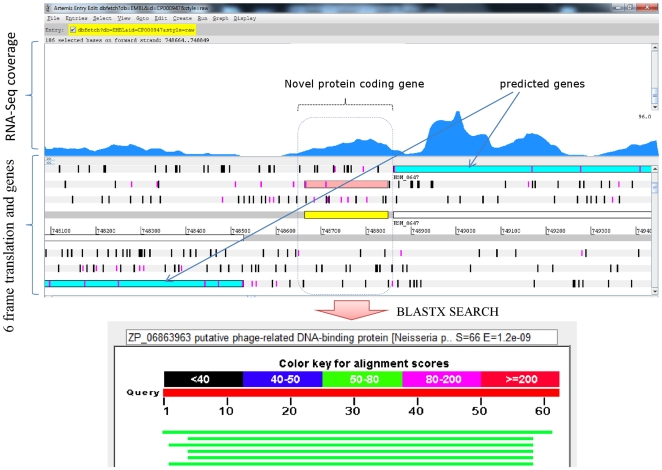
Identification of a novel protein coding gene. Novel protein coding gene “HSP7” identified using transcriptome analysis shows homology (similarity 74%, sequence coverage 100%) to a phage related DNA binding protein from *Neisseria polysaccharea.*

We evaluated the coding potential of all expressed intergenic regions, by conducting BLASTX based sequence searches against the non-redundant protein database at NCBI followed by manual analysis and interpretation. We identified 38 novel protein coding regions (Table 2). The average length of the identified novel proteins was around 60 amino acids (ranged from 19 to 135 amino acids). The majority of the novel proteins (30) were conserved hypothetical proteins present in related species such as *H. somni* 129PT, *M. haemolytica*, and *H. influenzae*. Some of the novel proteins had predicted functions, such as DnaK suppressor protein, toxic membrane protein TnaC, and predicted toxic peptide ibsB3 (Table 2). Figure 4 shows an example of a novel protein “HSP7” that is similar (74% similarity and 100% coverage) to a putative, phage-related DNA-binding protein of *Neisseria polysaccharea.*

**Table 2:** Novel proteins identified in the *H. somni* 2336 genome along with closest matching homolog and its annotation.

ID	Start	End	Strand	Length (nt)	Top BLASTX Hit	Annotation
HSP1	140988	141083	+	96	ZP_04978675.1	hypothetical protein MHA_2182 [*Mannheimia haemolytica* PHL213]
HSP2	260019	260078	−	60	YP_002791255.1	toxic membrane protein [*Escherichia coli str. K-12 substr.* MG1655]
HSP3	260229	260408	−	180	YP_001784202.1	hypothetical protein HSM_0870 [*Haemophilus somnus* 2336]
HSP4	260707	260850	−	144	YP_001784202.1	hypothetical protein HSM_0870 [*Haemophilus somnus* 2336]
HSP5	260951	261007	−	57	CBY77851.1	predicted toxic peptide IbsB3 [*Escherichia coli BL21*(DE3)]
HSP6	692328	692543	−	216	ZP_04977604.1	hypothetical protein MHA_1062 [*Mannheimia haemolytica* PHL213]
HSP7	748664	748849	+	186	ZP_06863963.1	putative phage-related DNA-binding protein [*Neisseria polysaccharea* ATCC 43768]
HSP8	752366	752593	+	228	ZP_01791588.1	hypothetical protein CGSHiAA_00240 [*Haemophilus influenzae* PittAA]
HSP9	753226	753384	+	159	ZP_05848096.1	conserved hypothetical protein [*Haemophilus influenzae* RdAW]
HSP10	754234	754398	+	165	NP_873053.1	hypothetical protein HD0492 [*Haemophilus ducreyi* 35000HP]
HSP11	758474	758698	+	225	ZP_05848108.1	conserved hypothetical protein [*Haemophilus influenzae* RdAW]
HSP12	764501	764686	+	186	ABX51978.1	hypothetical protein [*Haemophilus phage* SuMu]
HSP13	771653	771787	+	135	ZP_04464387.1	hypothetical protein CGSHi6P18H1_07995 [*Haemophilus influenzae* 6P18H1]
HSP14	782712	782840	+	129	YP_001344686.1	hypothetical protein Asuc_1392 [*Actinobacillus succinogenes* 130Z]
HSP15	858416	858721	+	306	ZP_04976950.1	hypothetical protein MHA_0367 [*Mannheimia haemolytica* PHL213]
HSP16	982362	982619	+	258	NP_660225.1	repressor-like protein [*Haemophilus influenzae* biotype aegyptius]
HSP17	1008333	1008518	+	186	YP_001784474.1	hypothetical protein HSM_1144 [*Haemophilus somnus* 2336]
HSP18	1014064	1014276	+	213	ZP_02478185.1	hypothetical protein HPS_04457 [*Haemophilus parasuis* 29755]
HSP19	1023854	1024255	+	402	YP_719605.1	hypothetical protein HS_1393 [*Haemophilus somnus* 129PT]
HSP20	1026199	1026414	+	216	YP_002475212.1	putative lytic protein Rz1, bacteriophage protein [*Haemophilus parasuis* SH0165]
HSP21	1031209	1031379	+	171	YP_002475190.1	hypothetical protein HAPS_0589 [*Haemophilus parasuis* SH0165]
HSP22	1031709	1031906	−	198	ZP_05731317.1	hypothetical protein Pat9bDRAFT_4634 [*Pantoea* sp. At-9b]
HSP23	1043942	1044073	+	132	ZP_02479029.1	hypothetical protein HPS_00455 [*Haemophilus parasuis* 29755]
HSP24	1306383	1306649	−	267	ZP_04976986.1	hypothetical protein MHA_0405 [*Mannheimia haemolytica* PHL213]
HSP25	1309667	1309807	−	141	ZP_01787689.1	hypothetical protein CGSHi22421_00792 [*Haemophilus influenzae* R3021]
HSP26	1324541	1324765	−	225	YP_002475146.1	DnaK suppressor protein/C4-type zinc finger protein, DksA/TraR family [*Haemophilus parasuis* SH0165]
HSP27	1345868	1346077	+	210	YP_001088372.1	putative conjugative transposon egulatory protein [*Clostridium difficile* 630]
HSP28	1448209	1448292	−	84	AAB96578.1	TnaC [*Haemophilus influenzae*]
HSP29	1747221	1747361	+	141	YP_002476351.1	hypothetical protein HAPS_1915 [*Haemophilus parasuis* SH0165]
HSP30	1750020	1750235	+	216	ZP_04752631.1	hypothetical protein AM305_05314 [*Actinobacillus* minor NM305]
HSP31	1852968	1853201	+	234	YP_718779.1	hypothetical protein HS_0567a [*Haemophilus somnus* 129PT]
HSP32	1959818	1959922	−	105	ZP_04977712.1	hypothetical protein MHA_1177 [*Mannheimia haemolytica* PHL213]
HSP33	1962885	1963013	+	129	ZP_05993368.1	hypothetical protein COI_2717 [*Mannheimia haemolytica* serotype A2 str. OVINE]
HSP34	1962985	1963176	−	192	ZP_05993369.1	hypothetical protein COI_2718 [*Mannheimia haemolytica* serotype A2 str. OVINE]
HSP35	1966085	1966366	−	282	ZP_04977704.1	hypothetical protein MHA_1169 [*Mannheimia haemolytica* PHL213]
HSP36	1977131	1977247	+	117	ZP_07538596.1	hypothetical protein appser10_8220 [*Actinobacillus pleuropneumoniae* serovar 10 str. D13039]
HSP37	2071545	2071745	−	201	YP_719865.1	hypothetical protein HS_1660 [*Haemophilus somnus* 129PT]
HSP38	2165733	2165906	−	174	YP_718223.1	hypothetical protein HS_0017a [*Haemophilus somnus* 129PT]

### Corrections made to the existing genome annotation

The single nucleotide resolution map described in this study enabled us to correct the start site for five genes based on the current genome annotation (Table 3). These genes were annotated as phospholipid synthesis protein, ribosomal protein S2, aconitate hydratase 2, peptide chain release factor 2, and DUF411, a protein of unknown function. Based on evidence from RNA-Seq data, we performed a BLAST comparison with other phylogenetically similar proteins to confirm the new gene boundaries (Table 3).

**Table 3:** Genes with revised coordinate information based on transcriptome map.

Gene id	Previous annotation (Start-End)	New corrected annotation (Start-End)
HSM_0031	24651–24929	24597–24929
HSM_0525	602547–603416	602547–603602
HSM_0789	909036–911534	909036–911642
HSM_1019	1164444–1165163	1164444–1165244
HSM_1729	1972283–1972600	1972283–1972765

### Non-functional start codons and frameshifts

The comparison of the transcriptome map of the *H. somni* genome with predicted proteins revealed the presence of frameshift mutations. Four genes have non-functional start codons, resulting in a predicted protein, truncated at the amino terminus (based on BLAST comparison with homologous proteins in other species), although full length mRNA was present. An example is presented for the gene “HSM_0748”, annotated as “Alpha-L-fucosidase” (Figure S1). The other three genes, HSM_0603, HSM_1666 and HSM_1668, encode a hypothetical protein, type III restriction protein res subunit, and CTP synthase, respectively. Two genes with frameshifts causing protein truncations (based on BLAST comparison with homologous proteins) are HSM_1385 (beta-hydroxyacyl dehydratase, FabA) and HSM_1744 (alcohol dehydrogenase zinc-binding domain protein). The transcriptome map revealed a full length mRNA for these two genes that code for truncated proteins.

### Gene expression and operon structures

Our transcriptome map of *H. somni* identified expression from 1636 (approximately 80%) of the predicted genes. The expressed genes were distributed evenly across all TIGRFAM functional categories (Table S1). The transcriptome map allowed identification of operon structures at a genome scale, critical for identifying co-expressed genes and for understanding coordinated regulation of the bacterial transcriptome. We identified co-expression for 452 pairs (total 730 genes) of *H. somni* genes (Table S2) that were transcribed together and constituted a minimal operon. By joining consecutive overlapping pairs of co-expressed genes, we identified 278 distinct transcription units (Table S3).

**Figure 5:**
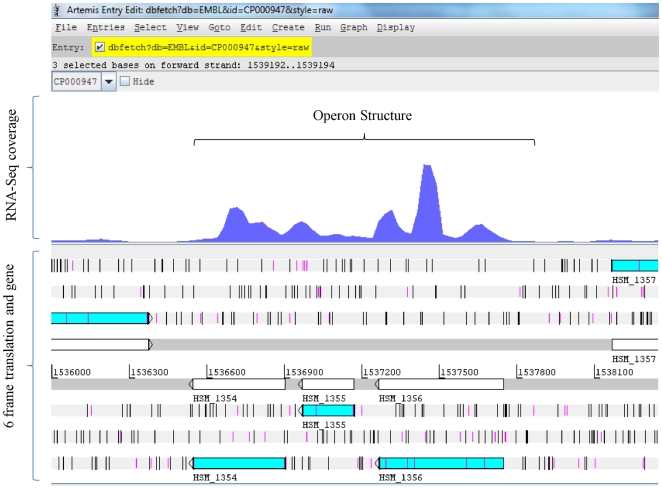
Identification of a novel operon structure comprised of three genes: HSM_1354, HSM_1355, and HSM_1356. The RNA-Seq coverage shows three genes annotated as ribosomal proteins (IF3, L35, and L20) being expressed as a transcription unit.

We compared our experimentally identified co-expressed genes with computationally predicted operons. The overlap between computational prediction of co-expressed genes using DOOR [24] and this study was 86% (394 gene pairs) (Table S4). Thus, our dataset validates expression of 394 computational gene-pair predictions. We identified 59 new gene pairs that are co-expressed and were not predicted by DOOR, which could be part of unidentified, new operon structures. For example, further in-depth analysis indicated a new operon consisting of three genes: HSM1354, HSM1355 and HSM1356, annotated as ribosomal protein L20, ribosomal protein L35, and translation initiation factor IF-3 respectively, which were not predicted computationally (Figure 5). The orthologs of these genes are well known to form a functional operon of ribosomal proteins (IF3-L35-L20) in *Escherichia coli* [25].

## Discussion

In this study using RNA-Seq we describe the whole genome transcriptome profile of *H. somni* 2336, a bovine respiratory disease pathogen. The single nucleotide resolution map helped uncover the structure and complexity of this pathogen's transcriptome and led to the identification of novel, small RNAs and protein coding genes as well as gene co-expression. Prokaryotic genome annotation is performed often using computational gene prediction programs [8], [9]. However, these prediction algorithms are not able to identify the non-coding sRNAs, antisense transcripts, and other small proteins. To overcome the shortcomings of computational genome structural annotation, various experimental methods are used for identification of novel expressed elements [13], [14], [15], [16], [17], [18], [26], [27], [28]. Deep transcriptome sequencing (RNA-Seq) has emerged recently as a method that enables the study of RNA-based structural and regulatory regions at the genome scale. RNA-Seq technology has many advantages compared with existing array based methods for transcriptome analysis. In particular, RNA-Seq does not require probes, so the process is free from probe design issues or bias from hybridization issues. Also, the transcriptome coverage from RNA-Seq is very high [29], [30]. RNA-Seq was demonstrated to be effective for the discovery of bacterial non- coding RNAs, accurate operon definition, and correction of gene annotation [27], [31], [32]. Therefore, in the current study, we used RNA-Seq for profiling *H. somni* 2336 transcriptome.

Mapping of RNA-Seq reads onto the *H. somni* genome sequence resulted in more than 94% coverage with at least one read per base. This observation is consistent with the reported 94% genome expression in *Bacillus anthracis*, 89.5% in *Sulfolobus solfataricus*, and 95% in *Burkholderia cenocepacia,* studied under one or more experimental growth conditions using RNA-Seq [32], [33], [34]. These results indicate that most of the bacterial genome sequence is expressed at some basal level. To identify significantly expressed regions above this baseline, we used two alternative methods (discussed in Results section) to estimate the background expression. Both methods yielded similar results (6–7 reads per base). We selected the higher stringency cutoff of 7 reads per base to minimize the number of false positives.

We identified a total of 95 sRNAs in the *H. somni* genome. Twelve of these were predicted by Rfam [23] and are similar to conserved sRNA (e.g., 6S, tmRNA, FMN) in other bacterial species, which helps validate our approach. The 83 novel *H. somni* sRNAs may have housekeeping function, regulatory activity, or participate in virulence as described in other pathogenic bacteria [19], [35], [36]. The identified sRNAs did not show any location specific bias across the genome. Similarly, genes known to be associated with virulence are known to be scattered across bacterial genomes [37], [38]. However, the tendency to form clusters was observed with sRNAs, which could indicate that functionally related sRNAs tend to be located in close proximity.

The RNA-Seq based transcriptome map of *H. somni* identified 38 novel protein coding genes that were missed by the initial annotation. The average length of the proteins coded by these genes exceeds 60 amino acids, suggesting that length based cutoff was not the main reason that these genes were missed by computational gene prediction programs. The novel protein coding genes identified in the current study could serve as a training set to improve gene prediction algorithms.

The transcriptome map helped to identify incorrect annotation of start codons in the genome. Transcriptional mapping does not provide direct evidence of translational start sites. However, location of identified transcriptional start sites suggest that the annotated start codons are incorrect, an observation that is confirmed by BLAST comparisons against homologous genes in other bacterial species. Transcriptional mapping revealed genes where the 5′ untranslated sequence extended well beyond the translational start. BLAST comparisons indicated that these genes have either nonsense or missense base changes relative to homologous genes in other bacterial species, causing apparent “truncated” proteins compared with those in other species. Further work is needed to determine whether these 5′ untranslated regions serve regulatory functions or they are vestigial.

RNA-Seq data enabled us to determine operon structures at a genome scale, and it allowed identification of some operons not predicted by the computational operon prediction method. Operon structures that include genes not expressed under the experimental growth condition used in the current study, could not be identified. Our results support the notion that using a combination of experimental operon identification by RNA-Seq and computational prediction can improve operon identification in bacterial genomes [39].

For the first time, we report the RNA-Seq based transcriptome map of *H. somni* 2336 and describe novel expressed regions in the genome. Whereas the results are interesting, we are aware of the limitations of the study. Because the RNA-Seq protocol was not strand specific, we could not determine the strand specificity of expressed novel transcripts. Therefore, Table 1 lacks information about sRNA orientation in the genome. Because strand specific information was missing, we could not describe antisense expression in the genome. For protein coding genes, we derived strand specificity based on alignment of the BLAST hit. Despite this shortcoming, we identified novel expressed regions and transcriptional patterns across the whole genome at a high coverage, which is not possible by other transcriptome analysis methods.

Overall, this study describes RNA-Seq based transcriptome map of *H. somni* for identification of functional elements in a pathogen of importance to agriculture. Our genome-wide survey predicts numerous, novel, expressed regions that need biological characterization for understanding disease pathogenesis. Description of all functional elements in the *H. somni* system is a prerequisite for conducting holistic systems approaches to understand the complex pathogenesis of bovine respiratory disease.

## Methods

### RNA isolation and sequencing

We propagated *H. somni* 2336 on three TSA-blood plates (with 5% sheep red blood cells) for 16 hr or until a fresh lawn of cells was visible. IBC approval was not required for acquiring the plates as they were purchased through a commercial vendor: Fisher Scientific (Pittsburgh, PA), and manufactured by Becton Dickinson Diagnostic Systems, (Franklin Lakes, NJ). We washed the plates with brain heart infusion (BHI) broth, adjusted the culture to an OD620 nm = 0.8, and supplemented with RNAprotect reagent. The cells were harvested by centrifugation and stored at −80°C. We extracted total RNA using the RNeasy mini kit (Qiagen, Valencia, CA) following the manufacturer's protocol. Total RNA was treated with RNase-free DNAse (Invitrogen, Carlsbad, CA). Using Bioanalyzer 2100 (Agilent Technologies, Santa Clara, CA), we determined the RNA integrity number (RIN) of total RNA to be greater than 8. MICROBExpress™ Kit (Ambion, TX, USA), which specifically removes rRNAs, was used for mRNA enrichment. Small RNAs (i.e., tRNA and 5S rRNA) are not removed with this enrichment step (confirmed by Bioanalyzer).

We used 100 ng enriched mRNA with Illumina mRNA-Seq sample preparation kit (Illumina, San Diego, CA) for library construction following the manufacturer's protocols. Briefly, mRNA was fragmented chemically by divalent zinc cations and randomly primed for cDNA synthesis. After ligating paired-end sequence adaptors to cDNA, we isolated fragments of approximately 200 bp by gel electrophoresis and amplified. We sequenced one nM of mRNA-Seq library on the Illumina GAII (Illumina, San Diego, CA), according to the manufacturer's protocol. Single read sequencing (36 bp) of the clustered flow cell was performed by Illumina's SBS chemistry (v3) and SCS data analysis pipeline v2.4. We used Illumina Real Time Analysis (RTA v1.4.15.0) software for flow-cell image analysis and cluster intensity. Subsequent base-calling was performed using the Illumina GA Pipeline v1.5.1 software.

### Mapping and analysis of Illumina reads

We checked all Illumina reads for quality, and removed sequence reads containing “Ns”. Custom perl script was written to convert Illumina reads into fastq format. The script “fq_all2std.pl” from MAQ [40] converted fastq format to Sanger fastq format. Reads in sanger fastq format, were mapped onto the *Histophilus somni 2336* genome sequence (GenBank Accession number. CP000947) using the alignment tool Bowtie [41], allowing for a maximum of two mismatches. The reads that mapped to more than one location were discarded. We used Samtools [42] to convert data into SAM/BAM format, and to generate alignment results in a pileup format. Pileup format provides the signal map file and has per-base format coverage. Custom perl scripts were written to calculate the background expression. Processed data was deposited in GEO with the accession number GSE29578.

### Analysis of intergenic regions of *H. somni* genome

We used in-house perl scripts to extract novel expressed intergenic regions to identify novel small RNAs, riboswitches, and putative novel proteins. sRNA <70 bp in length were discarded to minimize the number of false positives. For each novel expressed region, BLAST sequence searches were performed against the non-redundant protein database at NCBI to identify potential protein coding regions. Intergenic regions within predicted operons [24] represent expressed regions and can be mis-classified as sRNAs. Therefore, these regions were excluded. We analyzed BLAST results manually, to identify novel protein coding regions and start codon corrections. If no protein coding region was found in the intergenic expressed regions, the presence of a promoter or a rho-independent terminator allowed us to classify the regions as sRNA. Bacterial promoter sequences were predicted by Neural Network Promoter Prediction program (http://www.fruitfly.org/seq_tools/promoter.html) [43]. Rho-independent transcription terminators were identified using the program TransTermHP [44]. For functional annotation, all identified identified sRNA sequences were searched against the Rfam database [23]. sRNA sequence conservation among other genomes was determined by blastn searches against non-redundant nucleotide database at NCBI. We mapped sRNAs, along with additional features, onto genome browsers like IGV [45] and Artemis [46] for further visualization, manual analysis, and interpretation.

### Analysis of annotated regions of *H. somni* genome

*Gene expression:* expressed reads with coverage above background were mapped onto the annotated genes of H. *somni* 2336. Genes that had a significantly higher proportion of their length (>60%) covered by expressed reads were considered to be expressed.

*Operons:* RNA-Seq can identify and predict operon structures in bacteria. We considered two or more consecutive genes to be part of an operon, if they fulfilled the following criteria: (a) they are expressed; (b) they are transcribed in the same direction; and (c) the intergenic region between the genes is expressed. Overlapping pairs of such genes were joined together to identify large operon structures. We used in-house perl scripts for the analyses.

## Supporting Information

Figure S1 **Mutated start codon.** The Figure shows that the predicted protein coding frame (MH_748) is shorter at the 5′ end than the corresponding transcript level shown by the RNA-Seq coverage. Although the transcript is longer near 5′ end, no start codon is found in that region which might be a result of the mutation in that region of the start codon. This was further validated using homology searches of the full length transcript which shows high homology (95% Identity and >95% coverage) to a alpha-L-fucosidase protein from M. *haemolytica* PHL213.
(TIF)

Table S1 ***H. somni*** **genes expressed in the present study according to the TIGRFAM categories.**
(XLS)

Table S2 **Pairs of co-expressed genes identified in** ***H. somni 2336 genome*** **by RNA-Seq data analysis.**
(XLS)

Table S3 **Transcription units identified by joining co-expressed genes in** ***H. somni*** **2336.**
(XLS)

Table S4 **Comparison of co-expressed gene pairs identified from RNA-Seq data and operon prediction program “DOOR”.**
(XLS)
