# Supplementary figures and images for: RNA-Seq Based Transcriptional Map of Bovine Respiratory Disease Pathogen “Histophilus somni 2336”
Source: PLoS One. 2012 Jan 20;7(1):e29435. doi: 10.1371/journal.pone.0029435 (PMC3262788; doi:10.1371/journal.pone.0029435)

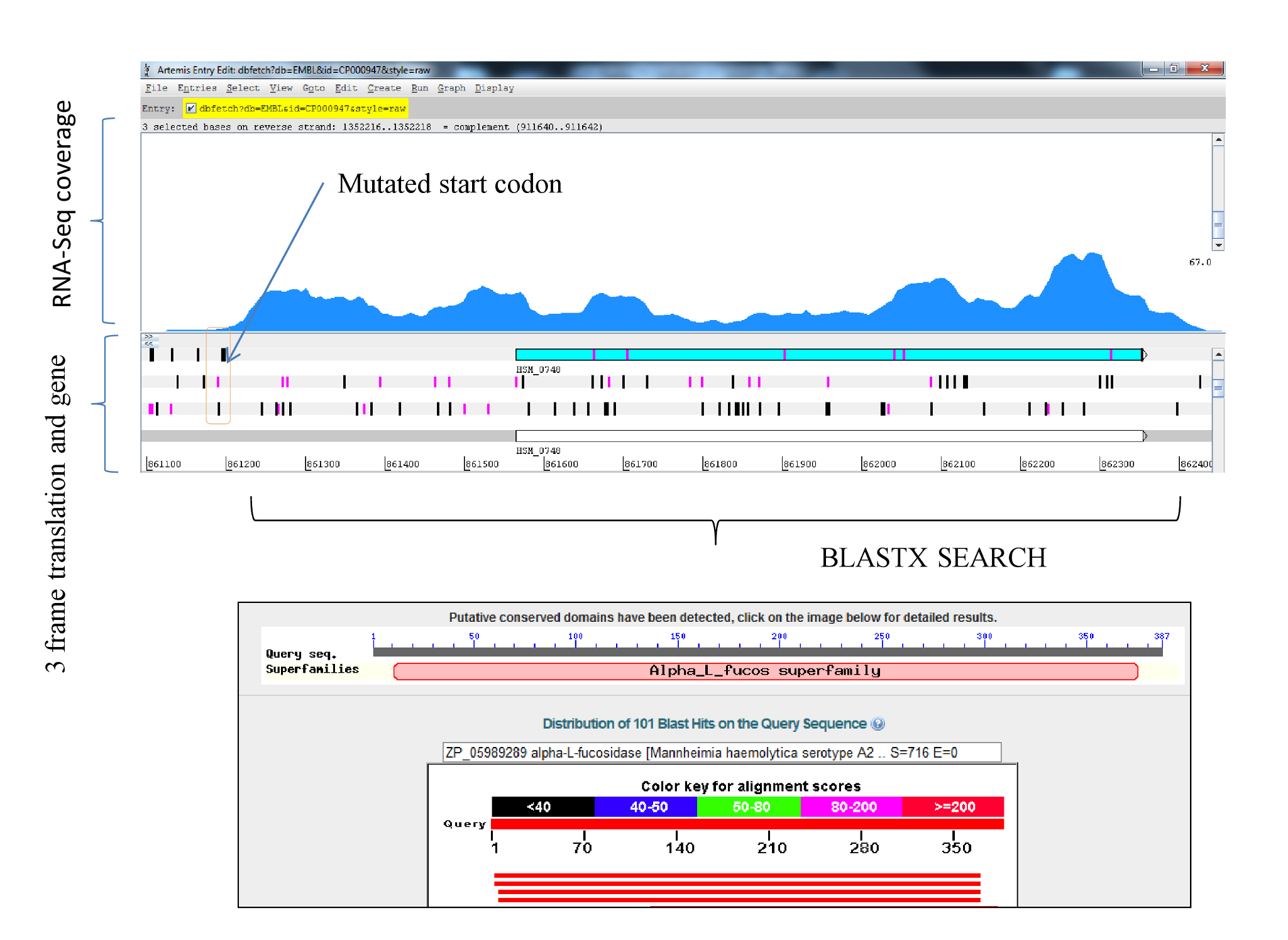

Supplement: Figure S1 — Mutated start codon. The Figure shows that the predicted protein coding frame (MH_748) is shorter at the 5′ end than the corresponding transcript level shown by the RNA-Seq coverage. Although the transcript is longer near 5′ end, no start codon is found in that region which might be a result of the mutation in that region of the start codon. This was further validated using homology searches of the full length transcript which shows high homology (95% Identity and >95% coverage) to a alpha-L-fucosidase protein from M. haemolytica PHL213. (TIF) [file pone.0029435.s001.tif]
